# Nonalcoholic Fatty Liver Disease for Identification of Preclinical Carotid Atherosclerosis

**DOI:** 10.1097/MD.0000000000002578

**Published:** 2016-01-22

**Authors:** Dong Hyun Sinn, Soo Jin Cho, Geum-Youn Gwak, Juhee Cho, Seonhye Gu, Donghyeong Seong, Danbee Kang, Hyunkyoung Kim, Byoung-Kee Yi, Seung Woon Paik

**Affiliations:** From the Department of Medicine, Samsung Medical Center, Sungkyunkwan University School of Medicine (DHS, G-YG, SWP); Center for Health Promotion, Samsung Medical Center (SJC); Department of Health Science and Technology, Samsung Advanced Institute for Health Science and Technology, Sungkyunkwan University (JC, DS, DK, B-KY); Department of Health, Behavior and Society and Epidemiology, Johns Hopkins Bloomberg School of Public Health, Baltimore, USA (JC); Biostatistics and Clinical Epidemiology Center, Samsung Medical Center (SG); Department of Otorhinolaryngology (HK); and Department of Medical Informatics, Samsung Medical Center, Seoul, South Korea (B-KY).

## Abstract

Nonalcoholic fatty liver disease (NAFLD) is associated with cardiovascular disease, yet whether identification of NAFLD in asymptomatic individuals is helpful over established risk factors remains unknown. A total of 37,799 asymptomatic adults aged 20 years or older who underwent comprehensive health check-up examination, including abdominal and carotid artery duplex ultrasonography (US) were included in the analysis. Nonalcoholic fatty liver disease was diagnosed with US and exclusion of secondary causes of fat accumulation or other causes of chronic liver disease, and graded as mild or moderate to severe fatty liver. Individuals with carotid plaque identified on carotid artery US were considered at risk for cardiovascular disease. Metabolic syndrome (MetS) was defined by the adult treatment panel III criteria. Nonalcoholic fatty liver disease was an independent factor associated with carotid plaque in a dose-dependent manner (odds ratio [OR]; 95% confidence interval [CI]: 1.09 [1.03–1.16] and 1.13 [1.06–1.21] for mild and ≥ moderate degree of NAFLD). Among clinically-relevant subgroups, NAFLD was more closely associated with carotid plaque in young adults (aged < 60 years) without MetS (OR [95% CI]: 1.13 [1.03–1.19] and 1.16 [1.06–1.27] for mild and ≥ moderate degree of NAFLD) than old adults (aged ≥ 60 years) or with MetS (OR [95% CI]: 1.06 [0.97–1.17] and 1.07 [0.97–1.19] for mild and ≥ moderate degree NAFLD). In young adults without MetS, the prevalence of carotid plaques was 32.8% and the sensitivity and specificity of NAFLD for carotid plaque was 0.38 and 0.67, respectively. In conclusion, NAFLD is associated with carotid plaque independent of traditional risk factors, especially in young adults without MetS. Nonalcoholic fatty liver disease could help identify additional individuals with preclinical atherosclerosis in asymptomatic young adults without MetS, yet, showed suboptimal performance as a screening tool.

## INTRODUCTION

Cardiovascular disease (CVD) is a major cause of morbidity and mortality worldwide.^1^ Early identification of the population at risk for CVD in the subclinical stage is of paramount importance to decrease the health care burden related to CVD. Currently, primary prevention efforts are aimed at identifying and managing individuals who have known risk factors for CVD such as metabolic syndrome (MetS) and smoking.^1^

Nonalcoholic fatty liver disease (NAFLD), characterized by accumulation of fat in the liver, is a chronic liver disease with rapidly increasing incidence that was proposed as the hepatic manifestation of metabolic derangement of the body.^2^ Nonalcoholic fatty liver disease is associated with an increased risk of CVD,^3^ raising the possibility that NAFLD might be a useful marker for assessing CVD risk. Nonalcoholic fatty liver disease and MetS, however, share many clinical features, such as obesity, dyslipidemia, and type 2 diabetes mellitus,^4,5^ with insulin resistance as a common pathophysiological mechanism. It is well established that MetS increases the risk of developing CVD and the progression of carotid atherosclerosis.^6^ Hence, it remains unclear whether identifying NAFLD in asymptomatic individuals is of further benefit over identifying MetS for differentiating the at-risk population for CVD.

This study aimed to assess the associations among NAFLD, MetS, and the risk of CVD, and to determine whether identifying NAFLD in asymptomatic individuals is helpful over identifying MetS for estimating the risk of CVD. We used the presence of carotid plaque as a surrogate marker for CVD risk, as carotid plaque is a good clinical model of early atherosclerosis^7^ and indicative of increased CVD risk.^8^

## METHODS

### Study Population

**FIGURE 1:**
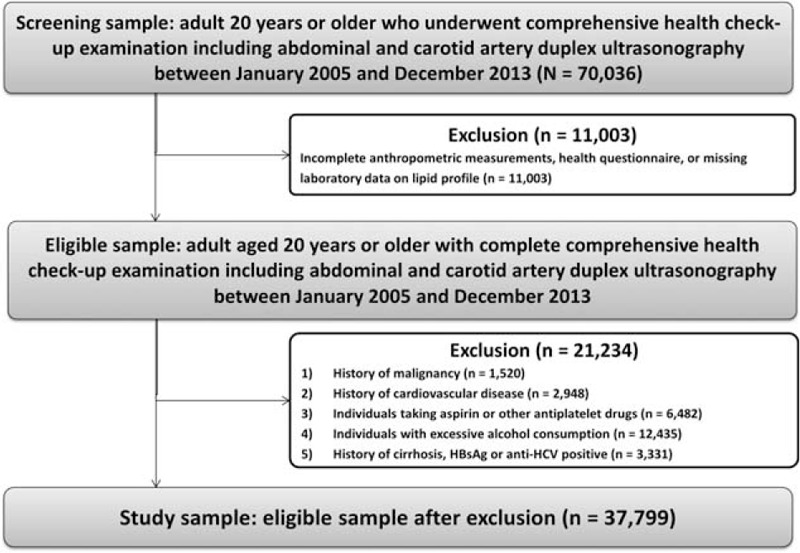
Flow diagram of the study participants.

We screened a total of 70,036 participants aged 20 or older who underwent a routine health check-up examination that includes abdominal ultrasonography (US) and carotid artery duplex US at the Center for Health Promotion of Samsung Medical Center in Seoul, Korea, from January 2005 to December 2013. Among them, we first excluded 11,003 participants with incomplete anthropometric measurements, health questionnaire, or missing laboratory data on lipid profile. Then we further excluded a total of 21,234 individuals who met any of following exclusion criteria: those with history of malignancy (n = 1520), history of CVD (n = 2948), individuals taking aspirin or other antiplatelet drugs (n = 6482), individuals with excessive alcohol consumption (>30 g/d for men and >20 g/d for women) (n = 12,435) and history of cirrhosis, hepatitis B surface antigen or anti-hepatitis C virus positive (n = 3331), to define adults free from cardiovascular disease or other chronic liver disease (Figure 1). The final study population included 37,799 asymptomatic individuals without CVD or chronic liver disease. The study protocol was approved by the institutional review board of Samsung Medical Center. The requirement for informed consent was exempted by the institutional review board because the study was based on retrospective analysis of existing administrative and clinical data.

### Study Variables

Detailed description of study variables are provided in our previous article.^9^ In brief, study variables were acquired from a self-administered health questionnaire, detailed physical examination, laboratory values, abdominal US, and carotid duplex US.

Nonalcoholic fatty liver disease was defined when abdominal US revealed fatty liver, that was diagnosed based on known standard US criteria, including parenchymal brightness, liver-to-kidney contrast, deep beam attenuation, and bright vessel walls, and was graded on a scale of 0 to 2 (0 = none, 1 = mild, 2 = moderate to severe).^10–12^ The NAFLD fibrosis score (FS) was calculated exactly as described originally.^13^

Carotid plaque was defined as intima-media thickness >1.5 mm in any carotid segment in carotid duplex US.^7^ Intima-media thickness was measured in the internal carotid artery just distal to the carotid bifurcation.

Diagnosis of MetS was based on the presence of 3 or more of the components listed by the revised adult treatment panel III of the National Cholesterol Education Program,^14^ with modification of the criteria for abdominal obesity for the Korean population as suggested by the Korean Society for the Study of Obesity.^15^ Underweight, normal weight, overweight, and obesity was defined as body mass index (BMI) (kg/m^2^) <18.5, ≥18.5 and <23, ≥23 and <25, and ≥25, respectively, using the cutoff values for Asians.^16^

### Statistical Analysis

Difference between individuals with and without carotid plaque was compared using *t* test or χ^2^ test, as appropriate. The logistic regression analysis was performed to see whether NAFLD is independently associated with carotid plaque. In the multivariable logistic regression model, age, sex, BMI, smoking status, MetS, and NAFLD were included. Receiver operating characteristic (ROC) analysis was performed for MetS, NAFLD, ≥ moderate NAFLD, NAFLD with high FS to calculate area under ROC. For NAFLD with high FS, cutoff point was chosen from ROC analysis as there is no known cutoff value for NAFLD FS for carotid plaque. Sensitivity, specificity, positive predictive value, and negative predictive value were calculated. *P* values < 0.05 were considered significant.

## RESULTS

### Baseline Characteristics

The baseline characteristics of analyzed subjects are shown in Table 1. Those with carotid plaque were older, and more likely to have overweight or obesity (Table 1). Each of the MetS components, MetS, and NAFLD were more frequently observed in individuals with carotid plaque. The NAFLD FS was also higher in those with carotid plaque. Current smoking status was less frequently observed in individuals with carotid plaque; mean age, however, was significantly different by smoking status (54.5 ± 10.0, 52.6 ± 8.3, and 48.9 ± 8.5 years for nonsmoker, past smoker, and current smoker, respectively, *P* < 0.001). When individuals were grouped according to age, carotid plaque was more frequently observed in past or current smokers than nonsmokers: 29.5%, 37.2%, and 32.2% for non, past, and current smoker (*P* < 0.001) for age <60 years; 55.7%, 67.3%, and 69.8% for past and current smoker (*P* < 0.001) for age ≥60 years).

**TABLE 1:**
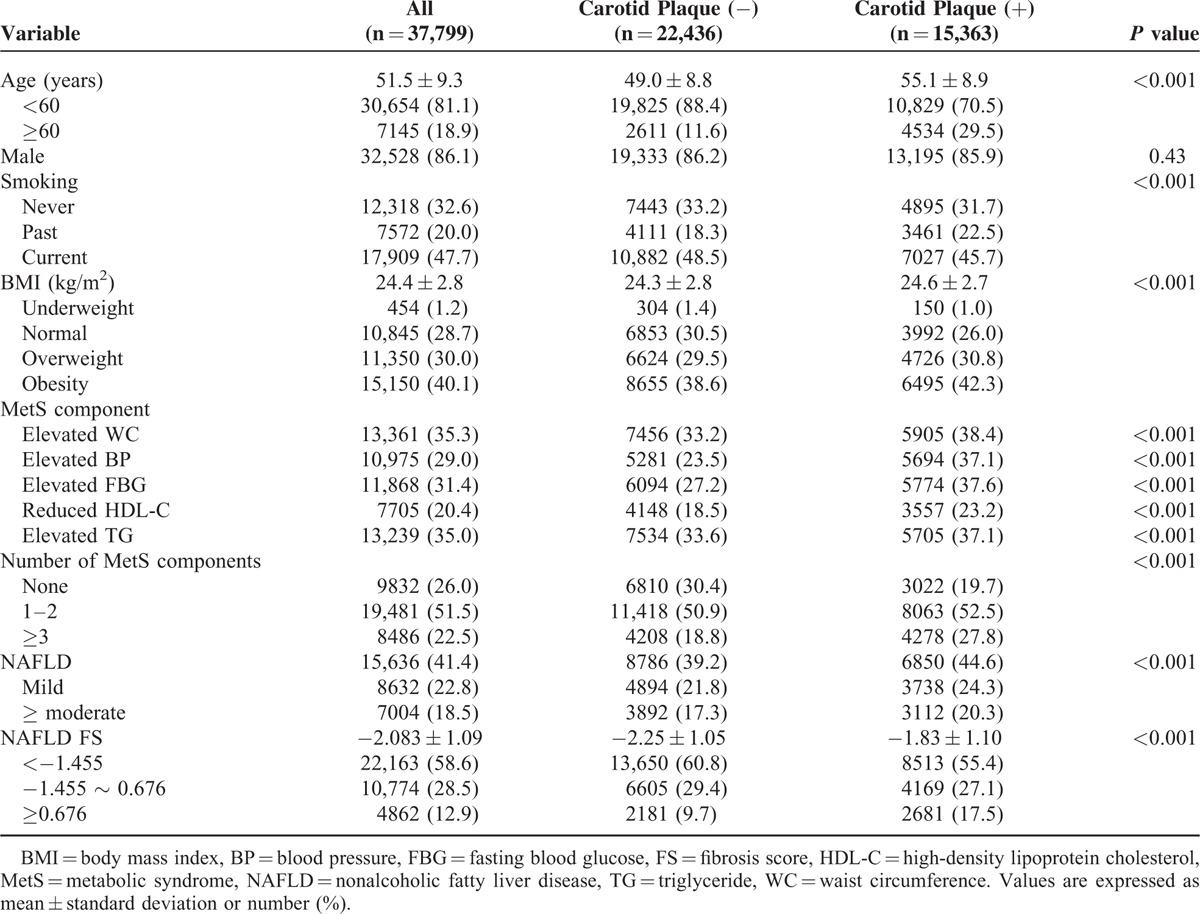
Baseline Characteristics

### Factors Associated with Carotid Plaque

**FIGURE 2:**
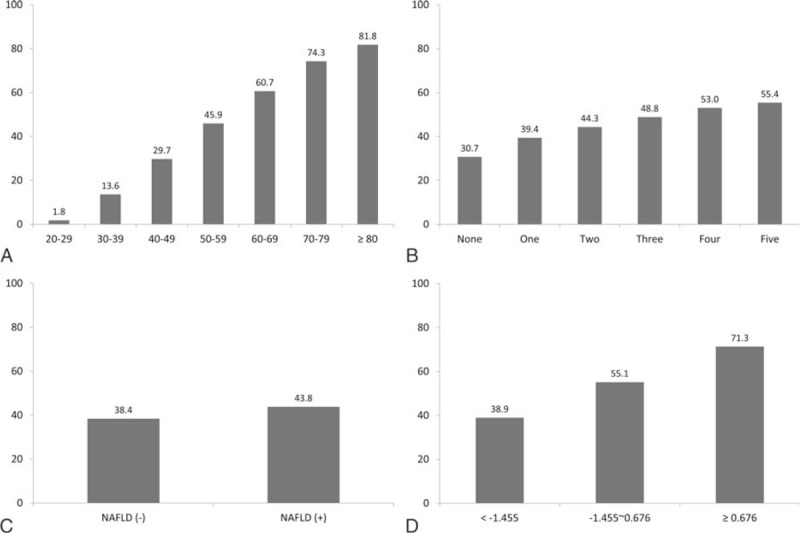
A, The prevalence of carotid plaques by age group. B, Number of metabolic syndrome components. C, Presence of nonalcoholic fatty liver disease. D, Nonalcoholic fatty liver disease fibrosis score. Carotid plaque was more frequently observed in adults with old age, those with metabolic syndrome, nonalcoholic fatty liver disease, and high nonalcoholic fatty liver disease fibrosis score.

The prevalence of carotid plaques was 40.6% (15,363/37,799). Age, male sex, BMI, smoking status, number of MetS components, and NAFLD were independent factors associated with carotid plaque (Table 2). There was also a dose-dependent relationship between NAFLD degree and carotid plaque [odds ratio (OR) (95% confidence interval (CI)): 1.09 (1.03–1.16) and 1.13 (1.06–1.21) for mild and ≥ moderate degree of NAFLD]. The prevalence of carotid plaque increased with increasing age (1.8%, 13.6%, 29.7%, 45.9%, 60.7%, 74.3%, and 81.8% for age 20–29, 30–39, 40–49, 50–59, 60–69, 70–79, and ≥80 years, respectively, *P* < 0.001, Figure 2A), and with increasing number of MetS components (30.7%, 39.1%, 44.3%, 48.8%, 53.0%, and 55.4% for 0, 1, 2, 3, 4 and 5 MetS components, respectively, *P* < 0.001, Figure 2B). The prevalence of carotid plaque was higher in those with NAFLD (43.8% vs. 38.4%, *P* < 0.001, Figure 2C). In patients with NAFLD, NAFLD FS was associated with carotid plaque (OR [95% confidence interval]: 1.46 [1.42–1.50], *P* < 0.001), with a prevalence of 38.9%, 55.1%, and 71.3% for NAFLD FS < −1.455, −1.455 to 0.676, and ≥0.676, respectively (Figure 2D, *P* < 0.001). The optimal cutoff value of NAFLD FS for carotid plaque was −2.075. The prevalence of carotid plaque was higher in those with high NAFLD FS (≥−2.075) than in those with low NAFLD FS (<−2.075) (50.8% vs. 36.7%, *P* < 0.001).

**TABLE 2:**
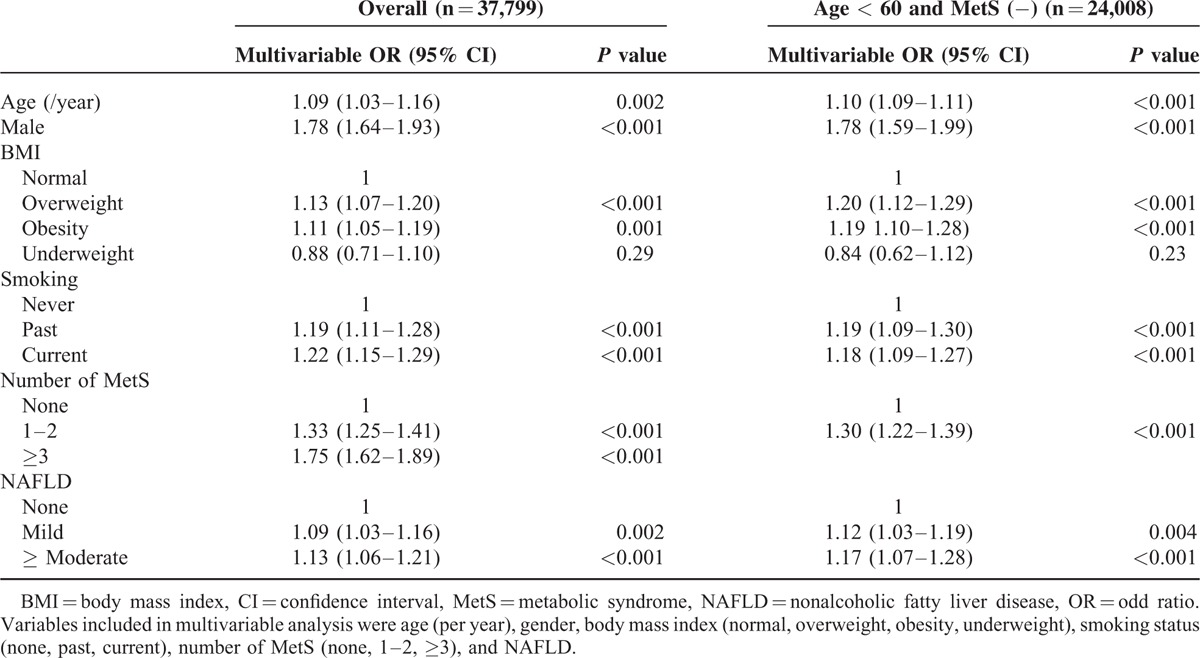
Factors Associated with Carotid Plaque

### Nonalcoholic Fatty Liver Disease and Carotid Plaque in Subgroup Analysis

**FIGURE 3:**
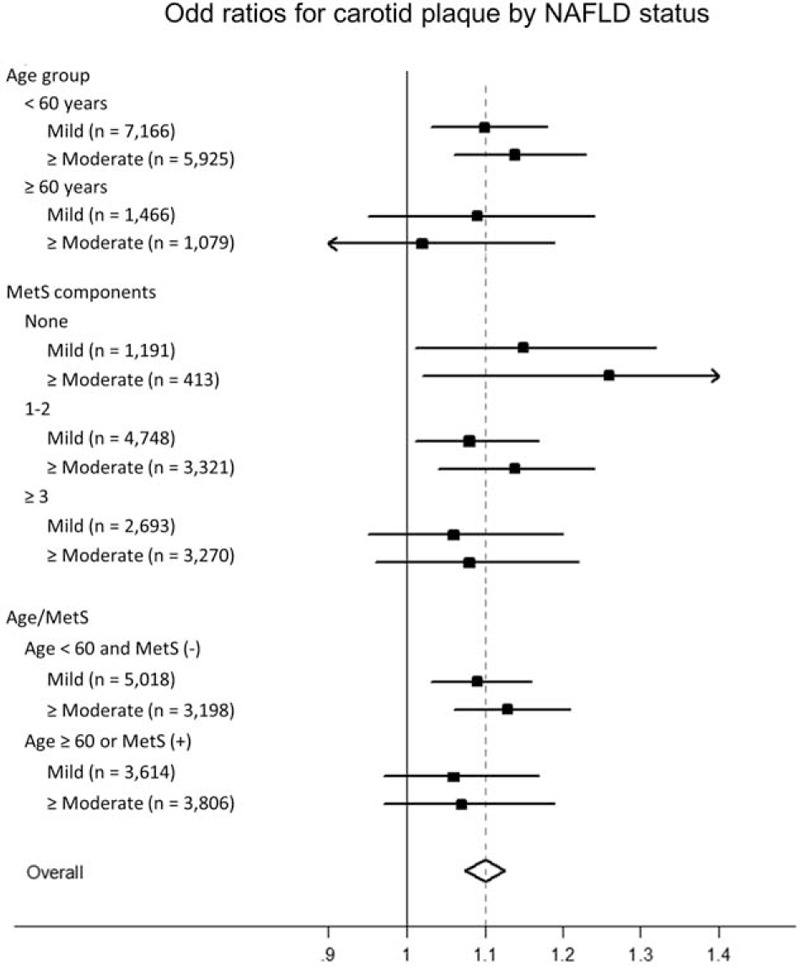
Association between carotid plaque and non-alcoholic fatty liver disease by clinically relevant subgroup. The association between carotid plaque and nonalcoholic fatty liver disease was more evident for those with young adults and those without metabolic syndrome.

Age was strongly associated with carotid plaque. Nonalcoholic fatty liver disease was also associated with age, and was more frequently observed in individuals aged <60 years than ≥60 years (42.7% vs. 35.6%, *P* < 0.001). Among participants with carotid plaque, mean age of those with NAFLD were younger than those without NAFLD (54.0 ± 8.6 vs. 56.0 ± 9.0, *P* < 0.001). The association between NAFLD and carotid plaque was more prominent in young adults (aged <60 years; OR [95% CI]: 1.10 [1.03–1.18] and 1.14 [1.06–1.23] for mild and ≥ moderate degree of NAFLD), than in older adults (aged ≥ 60 years; OR [95% CI]: 1.09 [0.95–1.24] and 1.02 [0.87–1.19] for mild and ≥ moderate degree of NAFLD) (Figure 3). Nonalcoholic fatty liver disease was closely associated with MetS. The prevalence of NAFLD was high (70.3%) in individuals with MetS whereas it was low (16.3%) in individuals without any MetS component; the rate was in between (41.4%) for those with 1 or 2 MetS component (*P* < 0.001). The association between NAFLD and carotid plaque was more prominent in individuals without any MetS components (OR [95% CI]: 1.15 [1.01–1.32] and 1.26 [1.02–1.58] for mild and ≥ moderate degree of NAFLD) than in individuals with one or two MetS components (OR [95% CI]: 1.08 [1.01–1.17] and 1.14 [1.04–1.24] for mild and ≥ moderate degree NAFLD), or in individuals with MetS (OR [95% CI]: 1.06 [0.95–1.20] and 1.08 [0.96–1.22] for mild and ≥ moderate degree of NAFLD). When further stratified by age and MetS status, significant association between NAFLD and carotid plaque was observed for young adults without MetS (OR [95% CI]: 1.13 [1.03–1.19] and 1.16 [1.06–1.27] for mild and ≥ moderate degree of NAFLD), whereas the association was not statistically significant for old adults or those with MetS (OR [95% CI]: 1.06 [0.97–1.17] and 1.07 [0.97–1.19] for mild and ≥ moderate degree NAFLD).

### Nonalcoholic Fatty Liver Disease for Identifying the At-Risk Population for Cardiovascular Disease

Overall, the sensitivity and specificity of MetS for carotid plaque was 0.27 and 0.81, respectively (Table 3). Compared with MetS, NAFLD had higher sensitivity (0.44), but lower specificity (0.60), for carotid plaque. Addition of NAFLD to MetS improved sensitivity (0.53), but with a tradeoff in specificity (0.55). When stratified according to NAFLD severity, ≥ moderate degree of NAFLD or NAFLD with high FS (≥−2.075) showed high specificity for carotid plaque (0.82–0.83), but limited sensitivity (0.20–0.26).

**TABLE 3:**
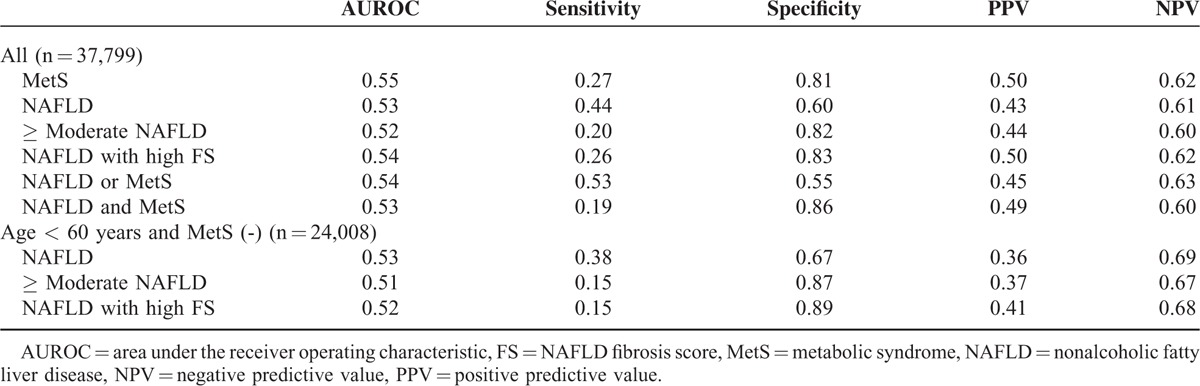
Performance of Nonalcoholic Fatty Liver Disease in Identifying Individuals with Carotid Plaque

In young adults without MetS, the overall prevalence of carotid plaque was 32.8%. Age, male sex, BMI, smoking, presence of 1 or 2 MetS components, and NAFLD status were risk factors for carotid plaque (Table 2). The sensitivity and specificity of NAFLD for carotid plaque was 0.38 and 0.67, respectively. A ≥ moderate degree of NAFLD or NAFLD with high FS showed high specificity (0.87–0.89) but low sensitivity (0.15) in this population.

## DISCUSSION

Ideal cardiovascular health is defined by the presence of both ideal health behaviors (nonsmoking, BMI < 25 kg/m^2^, physical activity at goal levels, and healthy diet) and ideal health factors (low total cholesterol, blood pressure, and fasting blood glucose).^1^ Individuals with unhealthy behaviors (smoking, obesity/overweight) or unhealthy factors (high cholesterol, blood pressure, and fasting blood glucose) can be considered at increased risk for CVD, and appropriate efforts (eg, smoking cessation, weight reduction, exercise, and aggressive management of MetS) should be taken to prevent the first occurrence of a clinical event.^1^ Thus, revealing and evaluating risk factors for CVD in the preclinical stage is of paramount importance to decrease morbidity and mortality related to CVD. In this study, old age, male sex, overweight/obesity, current or past smoking, and presence of MetS were independent factors associated with carotid plaque. Along with these well-known risk factors, NAFLD was another significant factor for carotid plaque. Likewise in previous studies,^17,18^ it was demonstrated that NAFLD is not only associated with hepatic prognosis, but also with increased risk of CVD. Furthermore, the association between NAFLD and carotid plaque was stronger for ≥ moderate degree NAFLD than mild degree NAFLD, showing a dose-dependent association. This finding is also in line with previous studies that demonstrated association between quantity of liver fat in NAFLD and the risk for CVD.^12,19,20^

Age was a prominent factor for carotid plaque. The prevalence of carotid plaque was only 1.8% among individuals aged 20 to 29 years, but increased gradually with age to over 80% for those aged ≥ 80 years. Among participants with carotid plaque, those with NAFLD were 2 years younger than those without NAFLD, indicating accelerated atherosclerosis in individuals with NAFLD. Previous study also indicated that carotid disease was found to occur approximately 8 to 9 years earlier in fatty liver patients than those without fatty liver.^21^ In addition, the association between NAFLD and carotid plaque was more evident among young adults in this study.

Metabolic syndrome was another strong risk factor for carotid plaque, as well as a factor associated with NAFLD. There was a significant difference in the prevalence of NAFLD by MetS status (70.3%, 41.4%, and 16.3% for those with MetS, 1–2 MetS components, and no MetS components). Nonalcoholic fatty liver disease, however, is sometimes observed in individuals without MetS. Zhang et al.^22^ showed that NAFLD can precede MetS. Lonardo et al.^23^ suggested that NAFLD can be a precursor of the MetS. In our previous study that included 5878 nonobese, nondiabetic individuals, NAFLD was identified in 15.2% of individuals without any MetS components, 28.5% of individuals with one MetS component, and 48.0% of individuals with 2 MetS components.^24^ Also, NAFLD was associated with increased insulin resistance regardless of MetS status.^24^ Musso et al.^25^ also reported that NAFLD is tightly associated with insulin resistance in nonobese and nondiabetic individuals than adult treatment panel III criteria defined MetS, indicating that NAFLD may help identify individuals with increased cardiometabolic risk in nonobese, nondiabetic population. When the risk of carotid plaque was further stratified according to the MetS status, the association between NAFLD and carotid plaque was more evident in individuals without any MetS components, followed by individuals with 1 or 2 MetS components. The association between NAFLD and carotid plaque was weak for individuals with MetS. Added to the current knowledge, these finding suggest that NAFLD can be observed in apparently metabolic-healthy individuals (eg, individuals without MetS) and is not a harmless condition. Nonalcoholic fatty liver disease in adults without MetS may be indicative of increased CVD risk.

Regarding traditional risk factors, young adults without MetS are considered to be at relatively low risk for CVD. The prevalence of carotid plaque, however, was not low (32.8%) among young adults without MetS. Therefore, new screening tools to estimate CVD risk are required for this population, and NAFLD might be useful in this regard. To investigate the performance of NAFLD, we evaluated sensitivity, specificity, positive predictive value, and negative predictive value of NAFLD for carotid plaque (Table 3). Overall, NAFLD was more sensitive than MetS for carotid plaque, but was less specific. When NAFLD was further subdivided according to the severity, ≥ moderated degree of NAFLD or NAFLD with high FS was specific for carotid plaque, but was not sensitive. Presence of NAFLD plus MetS was more specific than MetS alone, but sensitivity was low. The diagnostic performance of NAFLD was not improved significantly for young adults without MetS, but NAFLD could identify additional patients with carotid plaque with a sensitivity of 0.38 and specificity of 0.67. A ≥ moderate degree of NAFLD or NAFLD with FS was highly specific in this population (0.87–0.89), but sensitivity was low (0.15–0.17). Currently, screening for NAFLD is not recommended in the general population.^26,27^ Recently, Lonardo et al.^28^ suggested the screening of NAFLD for high-risk individuals (eg, individuals with MetS), at risk for developing NAFLD and its hepatic and extrahepatic complication. The current study has shown that NAFLD among young adults without MetS can help identify additional individuals who are at increased risk for CVD, who would have been misclassified as cardiovascular healthy individuals by traditional means. Limited sensitivity and specificity of NAFLD for carotid plaque, however, suggest that screening NAFLD in the general population to evaluate CVD risk cannot be advocated at present. In terms of prevention of CVD-related morbidity and mortality, there may be a certain group that may benefit from screening of NAFLD. Further studies, including cost-effective analysis,^29^ are needed to demonstrate whether identifying NAFLD in individuals without MetS is an effective strategy.

This study also demonstrated that NAFLD FS is associated with carotid plaque; individuals with high NAFLD FS were more likely to have carotid plaque. Consistent with this study, previous studies showed that NAFLD FS is associated with atherosclerosis or CVD outcome.^30,31^ Similarly in a study of genotype 1 chronic hepatitis C patients, hepatic fibrosis was associated with a high risk of early carotid atherosclerosis especially for patients ≤55 years.^32^ When individuals with NAFLD were subdivided according to the NAFLD FS, a high NAFLD FS showed higher specificity for carotid plaque compared with NAFLD alone, but with a tradeoff in sensitivity (Table 3). As NAFLD FS can be easily calculated, it can be used to improve specificity for carotid plaque among individuals with NAFLD. The role of NAFLD FS as a screening tool for the at-risk population for CVD, however, is questionable at the current time because of the low sensitivity.

There are some limitations in this work. First, NAFLD was diagnosed with US, that may lead to an incorrect diagnosis of NAFLD in 10% to 30% of cases.^11^ Nevertheless, US is the most frequently used tool for diagnosing NAFLD because of its noninvasiveness, acceptable accuracy, and wide availability, and is universally recommended by several guidelines on the diagnosis and management of NAFLD.^5,27^ We defined NAFLD by excluding individuals with chronic liver disease or with high alcohol consumption. Yet, this approach is limited to adequately rule out all the secondary form of steatosis, including steatosis from genetic alteration. Second, the analyzed subjects were all Koreans, mostly men, who visited the health center for a routine check-up. Health check-up participants are highly selected samples; hence the generalizability of this study to the general population, to women, or to other ethnicity needs to be demonstrated. Third, many patients were excluded from the analysis because of incomplete anthropometry and/or health check-up questionnaires, that may have introduced selection bias. Fourth, as this study is a cross-sectional analysis, longitudinal studies are needed to ascertain causality. Finally, whether early identification and intervention of NAFLD can actually decrease CVD mortality is still an open question.

In summary, this study demonstrated the association of NAFLD with subclinical atherosclerosis independent of established risk factors. Subgroup analysis showed that NAFLD is associated with carotid plaque in young adults without MetS. Limited sensitivity and specificity of NAFLD for carotid plaque suggest that screening NAFLD among young adults without MetS cannot be advocated at present; yet, NAFLD could identify additional patients with early atherosclerosis who would not be identified by traditional risk factors. Thus, NAFLD could be a valuable tool for assessing CVD risk in individuals without traditional risk factors that warrants further evaluations.
